# Supplementary material for: RNA polymerase backtracking results in the accumulation of fission yeast condensin at active genes
Source: Life Sci Alliance. 2021 Mar 26;4(6):e202101046. doi: 10.26508/lsa.202101046 (PMC8046420; doi:10.26508/lsa.202101046)
Supplement: Supplementary file 4 [file LSA-2021-01046_Supplemental_Data_1.docx]

**Supplementary Text**

*Simple model (Supplementary Fig S4A-B).* The model used to describe RNA elongation is a simple TASEP (Derrida *et al*, 1992). In this framework, flat, WT-like, profiles may be obtained by equating the speed rate of translocating agents and the termination rate ($\gamma_{term}=v_{c}$), the initiation rate ($\gamma_{init}$) controlling the global density of agents in the region (Supplementary Fig S4A). As already observed in (Brandão *et al*, 2019), profiles of residence time of condensin along the gene depend on the direction of condensin translocation (Supplementary Fig S4B). (1) When condensin and RNAP move in the same direction (full lines in Supplementary Fig S4B), head-to-tail collisions between both motors may occur. RNAPs then act as obstacles and slow down condensin movement. The shapes of the condensin residence profiles thus mostly follow the flat distribution of RNAP. The slowing-down of condensin is more important for genes denser in RNAPs (compare black & red full lines with blue & yellow full lines) and for lower bypassing rates (compare black & blue full lines with red & yellow full lines). (2) When condensin and RNAP move in opposite directions (dashed lines in Supplementary Fig S4B), head-to-head collisions occur. If the bypass rate of condensin over RNAP is fast enough, RNAPs represent mainly obstacles as in the head-to-tail case (black & blue dashed lines). For slow bypass rates (red & yellow dashed lines), RNAPs translocation forces condensin to move back leading to a dramatic gain in the residence time with increasing, super-linear, profiles along the gene. Regions denser in RNAPs show stronger effects. In both (head-to-tail, head-to-head) situations, residence profiles do not capture the overall shape of experimental condensin profiles observed in WT that exhibit a slow, gradual increase along the gene body followed by a strong accumulation closed to the transcription termination site (Fig 3).

*Backtrack model (Supplementary Fig S4C-I).* We assumed (i) that the backtracking rate is much stronger in the termination zone ($k_{back,min}\equiv k_{back}\left( gene body \right)=k_{back} (termination zone)/200$, Fig 4E), (ii) that the proportion of backtracked RNAPs (bRNAPs) in the gene body is very low ($k_{on}/k_{back,min}=100$) in WT, and (iii) that the effect of bRNAPs on the translocation of condensin is maximal ($v_{jump}^{b}=0$). Applying the same TASEP parameters as in the simple model ($\gamma_{term}=v_{c}=$0.4 s^-1^), we also observed flat profiles for RNAP (black lines in Supplementary Fig S4C) and we observed that bRNAPs mostly accumulate at the termination zone (red lines in Supplementary Fig S4C), with very similar behaviours whether the switching rates $k_{on}$ and $k_{back,min}$ are ‘fast’ (dashed lines) or ‘slow’ (full lines). However, the density of bRNAPs is not strong enough to strongly influence the translocation of condensins and the system mainly behaves like in the simple model (Supplementary Fig S4D), independently of the kinetics of switching. To increase the number of bRNAPs in the termination zone while maintaining a nearly flat profile for RNAP along the gene (with a minimal change in the parameters), we reduced the unbinding rate $\gamma_{term}$ to 0.2 s^-1^, stabilizing more bRNAPs at the termination zone (Supplementary Fig S4E). When the bypassing rate $v_{jump}^{m}$ over mobile RNAPs (mRNAPs) is fast enough, head-to-tail (black & blue full lines in Supplementary Fig S4F) and head-to-head (black & blue dashed lines) situations lead to similar shape in residence time with a flat or slightly increasing profile in the gene body (following the overall RNAPs density) followed by a sudden increased at the termination zone (as observed experimentally) due to the stronger speed reduction imposed by bRNAPs. When $v_{jump}^{m}$ is too small (red & yellow lines in Supplementary Fig S4F), the difference in bypassing rates between mRNAPs and bRNAPs is weak and the system behaves almost as in the simple model with low bypassing rates. Both behaviours (high vs low $v_{jump}^{m}$) persist for fast or slow switching rates (compare red & black with blue & yellow lines in Supplementary Fig S4F). Note that in order to observe a WT-like profile for condensin residence time, $v_{jump}^{b}$ does not necessarily need to be equal to zero, but the difference between $v_{jump}^{m}$ and $v_{jump}^{b}$ has to be large enough to differentiate the effect of mRNAPs and bRNAPs (green lines in the inset of Supplementary Fig S4F). In the model, the *tfs1DN* situation was mimicked by increasing the dwell time (${\sim1/k}_{on}$) of the backtracked state. In order to obtain a tilted RNAP profile as observed experimentally (Fig 3), we found that the switching rates $k_{on}$ and $k_{back}$ have to be ‘slow’ (compare black lines in Supplementary Fig S4G for ‘slow’ rates with black lines in Supplementary Fig S4H for ‘fast’ rates) and the increase in the life-time of the bRNAP state has to be strong enough ($k_{on}(WT)$/$k_{on}(tfs1DN)\gtrsim8$) (dotted and dashed black lines in Supplementary Fig S4G), as the tilted profile emerges from the formation of transient traffic jams generated by paused RNAPs with sufficiently large dwell-times. This leads to a significant increase of the bRNAPs density inside the gene body compared to the WT-like situation (red dashed and dotted lines in Supplementary Fig S4G). These changes in RNAP and bRNAPs profiles lead to the loss of the high condensin occupancy at the 3' of the gene and to an overall flattening of the residence time (black lines in Supplementary Fig S4I) if the stabilization of bRNAPs is not too strong and the corresponding RNAPs profiles are not too tilted (blue lines in Supplementary Fig S4I).

**Supplementary References.**

Brandão HB, Paul P, van den Berg AA, Rudner DZ, Wang X & Mirny LA (2019) RNA polymerases as moving barriers to condensin loop extrusion. *Proc Natl Acad Sci USA* 116: 20489–20499

Derrida B, Domany E & Mukamel D (1992) An exact solution of a one-dimensional asymmetric exclusion model with open boundaries. *J Stat Phys* 69: 667–687
